# Emergence of novel combinations of SARS-CoV-2 spike receptor binding domain variants in Senegal

**DOI:** 10.1038/s41598-021-02874-z

**Published:** 2021-12-08

**Authors:** Ambroise D. Ahouidi, Mary A. Rodgers, Abdou Padane, Nafissatou Leye, Ana Olivo, Moustapha Mbow, Aminata Mboup, Papa Alassane Diaw, Aminata Dia, Barbara Harris, Yacine Amet Dia Padane, Gora Lo, Todd V. Meyer, Cyrille K. Diedhiou, Diabou Diagne, Ndeye Coumba Toure Kane, Gavin Cloherty, Souleymane Mboup

**Affiliations:** 1 Institute for Health Research, Epidemiological Surveillance and Training (IRESSEF), Dakar, Senegal; 2 Abbott Global Surveillance Program, Abbott Laboratories, Abbott Park, IL USA

**Keywords:** Genetics, Diseases, Health care

## Abstract

The emergence of severe acute respiratory syndrome coronavirus 2 (SARS-CoV-2) lineages that carry mutations in the spike gene are of concern for potential impact to treatment and prevention efforts. To monitor for new SARS-CoV-2 mutations, a panel of specimens were sequenced from both wave one (N = 96), and wave two (N = 117) of the pandemic in Senegal by whole genome next generation sequencing. Amongst these genomes, new combinations of SARS-CoV-2 spike mutations were identified, with E484K + N501T, L452R + N501Y, and L452M + S477N exclusively found in second wave specimens. These sequences are evidence of local diversification over the course of the pandemic and parallel evolution of escape mutations in different lineages.

## Introduction

Ongoing viral evolution of severe acute respiratory syndrome coronavirus 2 (SARS-CoV-2) threatens the efficacy of our strongest defenses against coronavirus disease 19 (COVID-19): vaccines, therapeutics, and diagnostics. To keep pace with continual viral diversification, molecular surveillance serves as a critical alert system for identifying new strains to evaluate for potential immune or diagnostic escape. Most recently, the identification of SARS-CoV-2 lineages of concern, B.1.1.7 (alpha), B.1.351 (beta), P.1 (gamma), and B.1.617.2 (delta), immediately preceded their rise in prevalence and global spread^1–4^. Subsequent reports have demonstrated that increased transmissibility and immune escape are linked to these lineages, which are defined by spike receptor binding domain (RBD) mutations, including N501Y, K417N/T, L452R, and E484K. Notably, the E484K and L452R mutations in RBD had previously been demonstrated to confer immune escape in cell culture selection experiments^5^, which is consistent with their increasing prevalence^6,7^, possibly due to increased viral fitness^8,9^. Therefore, vigilant monitoring of circulating strains for these mutations is of critical importance for potentially preventing their spread.

**Figure 1:**
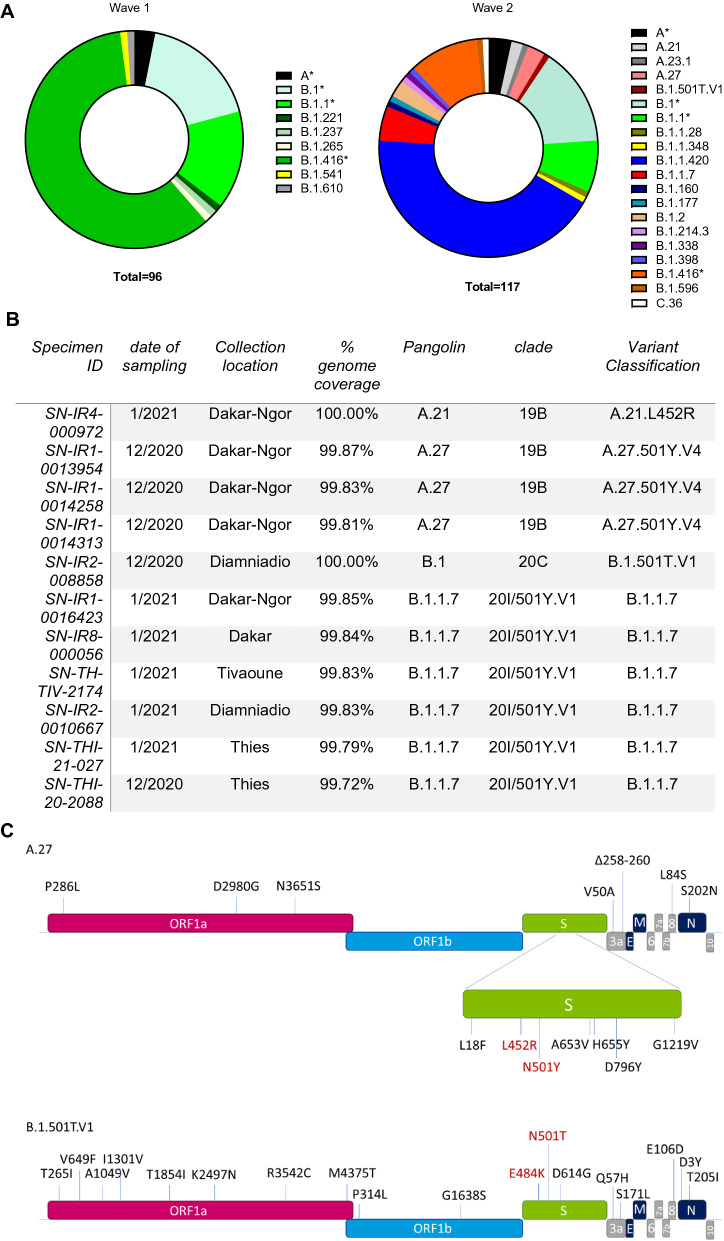
Molecular surveillance of SARS-CoV-2 in Senegal. In panel (**A**), the number of sequences classified in the indicated lineages present in waves one and two are shown proportionally to the total number of sequences generated with > 60% genome coverage from each wave as designated in the total numbers below each plot. An * indicates lineages that were present in both waves. In panel (**B**), the sequence and specimen metadata are listed for each strain carrying spike mutation of concern at position 501, 484, or 452. All sequences in this panel are from wave two. In panel (**C**), the lineage defining amino acid mutations (in comparison to the reference genome NC_045512) for the new strains identified in this study are shown. (**A**) Lineages identified, (**B**) Variant specimen summary, (**C**) Escape variant lineages.

The SARS-CoV-2 pandemic in Senegal has surged in several waves occurring in March–November of 2020 (wave 1), December 2020–March 2021 (wave 2) and July–September 2021 (Wave 3). The first variant of concern that was reported in Senegal was B.1.1.7, which was first identified in a patient who was diagnosed on December 30th, 2020 during the second wave^10^. To compare the SARS-CoV-2 strains circulating during the first two waves of the pandemic in Senegal, a panel of 150 first wave and 150 s wave leftover nasopharyngeal specimens in viral transport media (VTM) were collected in a study approved by the Ethical Committee of the Ministry of Health of Senegal (000129/MSAS/CNERS). VTM specimens were sequenced by next generation sequencing (NGS) using a metagenomic approach with probe enrichment (xGen) and analysis on an Illumina HiSeq^11^. Genomes were assembled using BLAST and sequence NC_045512 as a reference, followed by clade assignment and mutation analysis with the NextClade tool (clades.nextstrain.org) and lineage assignments with the Pangolin tool^12^. Genome coverage of > 60% was achieved for N = 213 specimens (N = 96 first wave, N = 117 s wave), with an average coverage depth of 43,006x (GISAID accession numbers EPI_ISL_1630259-1630270). The first wave genomes fell into 3 clades: 19B (N = 3), 20A (N = 78), and 20B (N = 15), similar to the composition of strains in other countries around the same time period^13^. In Pangolin nomenclature^14^, nine lineages were present in the first wave, which was predominated by B.1.416 (57/96, 59.4%, Fig. 1A). Viral diversity increased greatly in wave two with genomes from 9 clades present: 19A (N = 1), 19B (N = 11), 20A (N = 108), 20B (N = 81), 20C (N = 3), 20D (N = 1), 20E (N = 1), 20G (N = 1), and 20I (N = 1). Increased diversity of Pangolin lineages was also observed in the second wave, with 20 lineages identified, the majority of which were not present in the first wave (Fig. 1A). Most notable amongst the new strains found exclusively in wave two, the B.1.1.7 variant accounted for 5% of all second wave infections (6/117) and was present in four different cities (Dakar, Tivaoune, Diamnadio, and Thies, Fig. 1B), confirming a widespread distribution in western Senegal. The earliest B.1.1.7 infection in this study was diagnosed on December 21st in Thies, which predates the first case previously identified Senegal^10^. The December 21st patient was a patient who was tested due to contact with an infected person, suggesting that B.1.1.7 was already circulating in Senegal in early December. The remaining 5 B.1.1.7 cases were all diagnosed in early January during the exponential phase of the second wave spike in cases.

Escape mutations in the spike protein were absent from wave one but were present in 4% (5/117) of all wave two infections (Fig. 1B, Supplemental Table 1). Additional details for all genomes with mutations of concern at position 501, 484, and/or 452 in the spike RBD are shown in Fig. 1B. When classified by clade, all of the L452R mutations were exclusively found in 19B clade genomes whereas the L452M mutation appears to have emerged in wave two in the 20A clade (Supplemental Table 1). In addition to strains carrying L452R individually, variant strains carrying a combination of L452R + N501Y (3/117, 2.6%) were also identified. The N501Y mutation confers higher affinity for the ACE2 receptor and is present in several variants of concern (alpha, beta, gamma) while L452R is a signature escape mutation found in the delta and epsilon lineages that also increases infectivity^4,8,9,15,16^. The combination of both of these mutations in one strain is of concern for potential rapid spread of an immune escape variant. All three of the genomes carrying the L452R/N501Y combination belonged to the A.27 lineage (clade 19B) and did not encode the D614G mutation that predominates most global infections today. Likewise, the other lineage defining mutations for variants of concern were absent in the A.27 genomes, with the exception of L18F and H655Y, which are both present in the gamma lineage (Fig. 1C). While 13 common single nucleotide polymorphisms (SNPs) were identified for this lineage, each individual genome had unique SNPs as well, suggesting they were not transmission linked cases. The three patients who had A.27 infections were diagnosed in the Almadie district of Dakar in December 2020 and ranged in age from 36 to 55 (Fig. 1B).

In addition to the L452R + N501Y double mutant, a single genome was identified that carried a unique combination of E484K + N501T spike RBD mutations in a B.1 lineage genome (clade 20C) with D614G also present. This lineage has been provisionally named B.1.501T.V1 (Fig. 1C). The patient who was infected with this variant strain was a patient who was diagnosed in December 2020 in Diamniadio (Fig. 1B). While E484K confers escape from neutralizing antibodies^17,18^, the N501T mutation enhances the spike receptor binding domain (RBD) affinity for ACE2 in vitro and is predicted to enhance transmissibility, similar to N501Y^19,20^. Strains harboring N501T first emerged in August of 2020 in Northern Italy^6^ and the N501T mutation has been found recently in an emerging Brazilian lineage that differs from B.1.501T.V1^21^. Alarmingly, N = 2122 N501T strains were posted to GISAID from specimens collected in the months that followed the identification of this specimen in Senegal (January–April 2021) from countries in Africa, Europe, Asia, North America, and South America (GISAID, date of accession April 18th, 2021)^6^. Altogether, these trends suggest that convergent evolution around the world is leading to mutations at spike positions E484 and N501 in many lineages, suggesting a possible increased fitness for viruses carrying these mutations.

## Supplementary Information

Supplementary Information.
