# Supplementary material for: Emergence of novel combinations of SARS-CoV-2 spike receptor binding domain variants in Senegal
Source: Sci Rep. 2021 Dec 8;11:23644. doi: 10.1038/s41598-021-02874-z (PMC8655062; doi:10.1038/s41598-021-02874-z)
Supplement: Supplementary file 1 — Supplementary Information. [file 41598_2021_2874_MOESM1_ESM.docx]

Supplemental Table 1

|  |  | Wave 1 | | Wave 2 | |  |  |
| --- | --- | --- | --- | --- | --- | --- | --- |
| Lineage | Clade | N | % | N | % | Spike mutations |  |
| B.1.221 | 20A | 1 | 1.04 |  |  | V213L, D614G |  |
| B.1.237 | 20A | 1 | 1.04 |  |  | D614G |  |
| B.1.265 | 20A | 1 | 1.04 |  |  | D614G |  |
| B.1.541 | 20A | 1 | 1.04 |  |  | D614G, E1144D |  |
| B.1.610 | 20A | 1 | 1.04 |  |  | D614G |  |
| A | 19B | 3 | 3.13 | 4 | 3.42 | none |  |
| B.1.1 | 20B | 14 | 14.58 | 9 | 7.69 | D614G |  |
| B.1 | 20A | 17 | 17.71 | 18 | 15.38 | D614G |  |
| B.1.416 | 20A | 57 | 59.38 | 12 | 10.26 | D614G (L452M wave 2 only) |  |
| A.21 | 19B |  |  | 2 | 1.71 | D614N |  |
| A.23.1 | 19B |  |  | 1 | 0.85 | F157L, V367F, Q613H, P681R |  |
| A.27 | 19B |  |  | 3 | 2.56 | L18F, L452R, N501Y, A653V, H655Y, D796Y, G1219V |  |
| B.1.1.28 | 20B |  |  | 1 | 0.85 | D614G, V1176F |  |
| B.1.1.348 | 20B |  |  | 1 | 0.85 | D614G, G1167A |  |
| B.1.1.420 | 20B |  |  | 50 | 42.74 | L18F, N440K, D614G |  |
| B.1.1.7 | 20I |  |  | 6 | 5.13 | del69/70, del144/145, N501Y, A570D, D614G, P681H, T716I, S982A, D1118H |  |
| B.1.160 | 20A |  |  | 1 | 0.85 | S477N, D614G |  |
| B.1.177 | 20E |  |  | 1 | 0.85 | A222V, D614G |  |
| B.1.2 | 20C |  |  | 3 | 2.56 | D614G |  |
| B.1.214.3 | 20A |  |  | 1 | 0.85 | T95I, T478K, D614G, T716I |  |
| B.1.338 | 20A |  |  | 1 | 0.85 | Y380H, S477N, D614G |  |
| B.1.398 | 20B |  |  | 1 | 0.85 | T95I, D614G |  |
| B.1.596 | 20A |  |  | 1 | 0.85 | D614G |  |
| C.36 | 20D |  |  | 1 | 0.85 | D614G, Q677H |  |
| Total |  | 96 |  | 117 |  |  |  |
